# SoilFutures-BR: Bias-Corrected Soil Temperature Projections for Brazil

**DOI:** 10.1038/s41597-026-07500-z

**Published:** 2026-06-25

**Authors:** Dimaghi Schwamback, Bruna S. Aguiar, Jullian S. Sone, Abderraman R. A. Brandão, André S. Ballarin, Jarbas Honorio de Miranda, Edson Cezar Wendland

**Affiliations:** 1 Department of Hydraulics and Sanitation, São Carlos School of Engineering, University of São Paulo, CxP. 359, São Carlos, 13566-590 São Paulo Brazil; 2 Graduate Program in Environmental Science and Technology, Federal University of Technology - Paraná, Curitiba, Paraná 81280-340 Brazil; 3 Department of Biosystems Engineering, “Luiz de Queiroz” College of Agriculture (ESALQ/USP), Av. Pádua Dias n.11 LEB/ESALQ/USP, Piracicaba, 13418-900 Brazil; 4 Department of Economics, University of Waterloo, Waterloo, N2L 3G1 Canada

## Abstract

Soil temperature is a fundamental variable for the Earth system, yet it remains highly sensitive to climate change, particularly in tropical regions such as Brazil. Despite its importance, no bias-corrected soil temperature dataset based on the latest CMIP6 projections is currently available for the country. Climate projections are model-dependent and often exhibit systematic biases, which makes bias correction an essential step for subsequent applications. To address this gap, we present STEM-BR: Soil Temperature Under Climate Change for Brazil, a new gridded dataset of historical (1950–2014) and future (2015–2100) soil temperature derived from 15 CMIP6 General Circulation Models (GCMs) under the SSP2-4.5, SSP3-7.0, and SSP5-8.5 scenarios. The dataset was generated through systematic regridding, bias correction based on ERA5 dataset, and statistical refinement, and provides a monthly series at 0.25° × 0.25° resolution, including both raw and bias-corrected outputs. We applied the Quantile Delta Mapping (QDM) approach to correct biases in monthly time series of soil temperature at three depths (0.07, 0.28, and 1.00 m). The corrected dataset is available for both historical and future simulations and shows significant improvements in representing observed climatology. Bias correction reduces residual errors in long-term means and distribution tails to within ±10%, while preserving latitudinal, vertical, and climatic gradients. Seasonal cycles are more accurately reproduced, with reduced warm biases and narrower inter-model spread, particularly in dry climate classes where raw ensemble errors are largest. Future projections indicate consistent warming across Brazil, with increases of about +2 °C (mean) to +5 °C (95th percentile) under SSP2-4.5, and up to +5 °C (mean) to +10 °C (95th percentile) under SSP5-8.5 by 2100. Warming is strongest in the Northeast, North, and Central-West regions, while subsurface layers exhibit smoother but still significant changes. By reducing systematic biases and providing multi-model projections across depths and scenarios, SoilFutures-BR fills a critical data gap and offers a valuable resource to support climate impact assessments and adaptation strategies for agriculture, food security, and water resource management in Brazil.

## Background and Summary

Climate change poses significant challenges to agriculture, water supply, and hence, food security worldwide, and this is particularly true for Brazil with its extensive agricultural sector and diverse hydroclimate regimes. Recent studies indicate that while both air and soil temperatures are affected by a warming climate, soil temperature is often more vulnerable to rapid changes, particularly in low-latitude regions where the time of emergence of climate signals can be anticipated earlier^1^. Soil temperature is fundamentally linked to key agricultural production and productivity, as it governs root growth, nutrient mineralization, and soil moisture dynamics^2^. Moreover, increased soil temperatures may accelerate subsoil carbon loss not only deteriorating soil fertility but also contributing to climate change with greenhouse gasses emission from agricultural systems^3^. The capacity of soil to support productive agriculture is therefore contingent not only on atmospheric changes (e.g. air temperature and precipitation) but also on critical, yet more sensitive, changes in soil thermal regimes, making its accurate assessment strategic to safeguard global food security^1^. Shifts in temperature regimes, precipitation patterns, and extreme weather events have been widely documented to affect crop yield, water resources, and soil health in Brazil, thereby threatening both national and global food supplies^4,5^.

The use of global climate models (GCMs) to investigate climate change impacts on a wide range of critical environmental domains and societal needs, such as water and food security, is of paramount importance from a perspective of a terrestrial closed system, but they fail to capture the local topographical and land-use influences that are vital for accurately determining regional climate phenomena^1^. Regional climate models, on the other hand, play a substantial role in understanding regional and local impacts for their finer resolution scale and incorporation of local-scale processes in a dynamical downscaling. Nevertheless, these regional models are not always available since they usually demand significant computational demands and complex parameterizations that introduce additional uncertainties^6,7^. To fill this gap, statistically downscaled climate models are promising alternatives that have significantly enhanced the reliability of climate projections at regional scales^8,9^. Statistical downscaling refers to the use of statistics-based techniques to determine the relationship between large-scale climate patterns and observed local responses. Within these statistics techniques, bias correction and regridding methods are now routinely applied to refine coarse outputs from GCMs, thereby preserving regional and local-scale heterogeneity essential for agricultural applications^10^ for example. In addition, studies comparing statistical and dynamical downscaling methods have demonstrated that each approach can provide nuanced insights into regional variability in precipitation and temperature, thereby enhancing these inputs to climate impacts studies^9,11^. Using appropriate climate inputs is particularly critical when assessing soil temperature trends to investigate the two-way feedback loop of impacts between agriculture and climate change, as uncertainties in its projection can lead to misinformed adaptation strategies^12^.

**Fig. 1:**
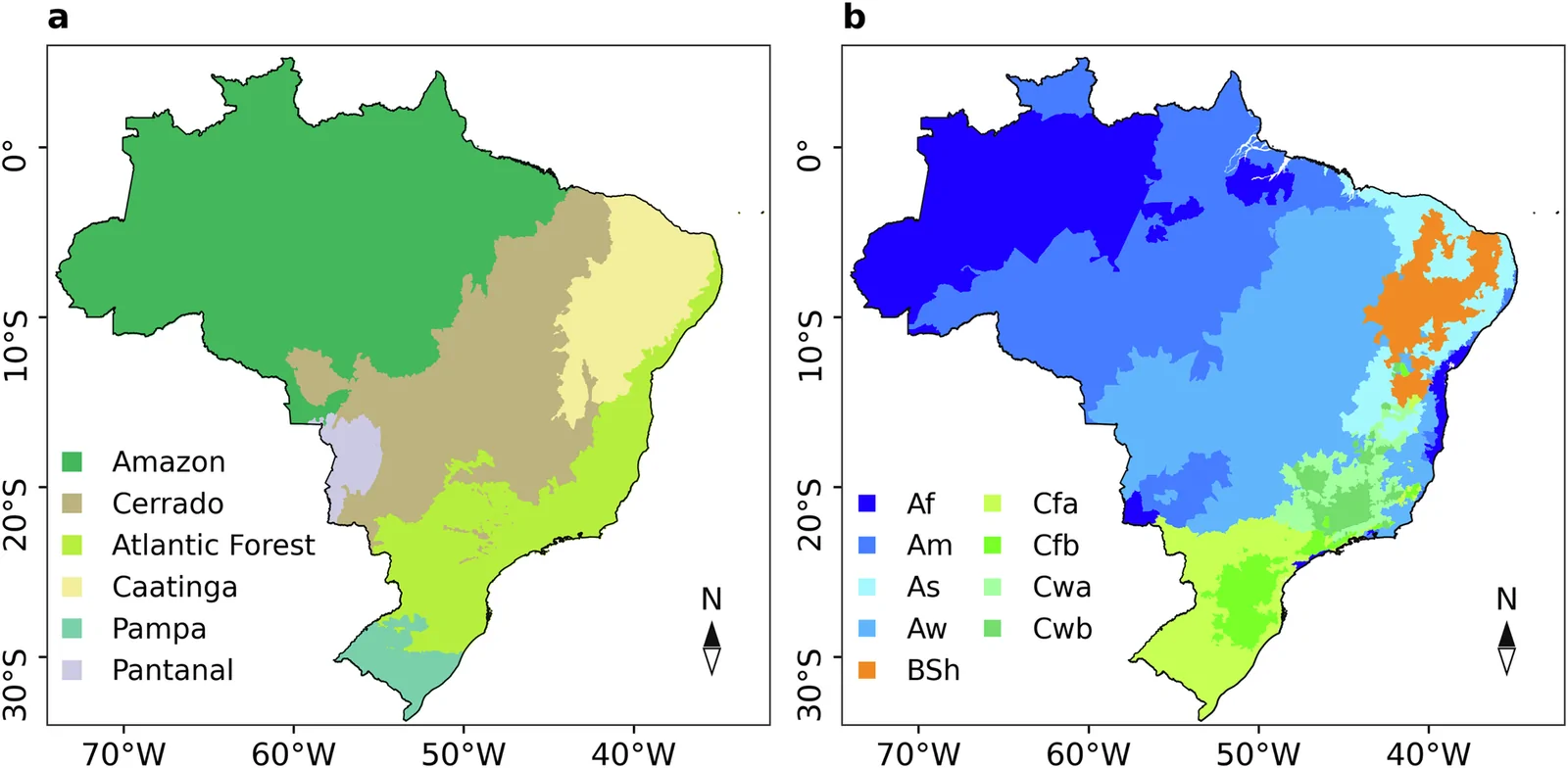
Biomes (subplot 1a) and climate zones (subplot 1b) of Brazil. Climate zones are classified according to the Köppen climate classification system: Af (Tropical rainforest), Am (Tropical monsoon), Aw (Tropical savanna), As (Tropical with dry summer), BSh (Hot semi-arid), Cfa (Humid subtropical), Cfb (Subtropical highland), Cwa (Humid subtropical with dry winter), and Cwb (Subtropical highland with dry winter).

Observed ground-based and remote-sensed soil temperature are usually scarce, especially in developing countries such as Brazil, and when it comes to validated finer-scaled projections, this limitation is drastically exacerbated, hindering landscape-oriented studies of soil processes. To the best of our knowledge, there is no bias-corrected downscaled soil temperature projections following the Shared Socioeconomic Pathways (SSPs) released with the Sixth Assessment Report (AR6) available for Brazil. In such a continental country, changes in soil processes forced by shifts in soil temperature have not only regional impacts but also affects the global climate and can cause global food supply disruptions, as increasing challenges faced by Brazilian agribusiness have been reported^13,14^. To fill this gap, we developed the SoilFutures-BR: a dataset containing bias-corrected statistically downscaled soil temperature projections based on three SSPs (SSP2-4.5, SSP3-7.0, and SSP5-8.5) covering the Brazilian territory (Fig. 1). A multi-model ensemble, as well as each 15 individual models, is easily available covering the historical (1950-2014) and future (2015–2100) periods for scientific and stakeholders’ use. SoilFutures-BR offers a crucial resource for enabling studies on climate change impacts on soil processes and how these processes change further impact vital uses of the soil, such as in agriculture, and contribute to exacerbate climate change.

## Methods

We developed the dataset described here by selecting 15 Global Circulation and Earth System Models (GCMs/ ESMs) within the Coupled Models Intercomparison Project Phase 6 (CMIP6) (Table 1). These models were selected based on their availability of monthly soil temperature (*tsl*) for the historical period (1950–2014) and future projections (2015–2100) under the *r1i1p1f1* variant and a maximum nominal spatial resolution of 250 km. The r1i1p1f1 member was used because it is it represents the first realization (r1), initial initialization (i1), first physics version (p1), and first forcing dataset (f1) in climate model ensembles, providing a standardized baseline that reduces variability caused by different initial conditions or model configurations, ensuring a homogeneous ensemble and avoiding unequal model weighting. Future projections were expressed in terms of the middle-of-the-road (SSP2–4.5), regional rivalry (SSP3-7.0), and fossil-fueled development (SSP5-8.5) Shared Socioeconomic Pathways, which describe alternative trajectories of future anthropogenic emissions and radiative forcing. The SSP2-4.5 corresponds to an intermediate pathway characterized by moderate socioeconomic development and mitigation efforts, leading to a stabilized radiative forcing level of 4.5 *Wm*^−2^ by 2100. On the other hand, the SSP3-7.0 represents a high-emission scenario associated with regionalized development, limited international cooperation, and continued dependence on fossil fuels, resulting in radiative forcing of 7.0 *Wm*^−2^. Lastly, the SSP5-8.5 depicts a worst-case, fossil-fueled development pathway, with rapid economic growth, energy-intensive lifestyles, and minimal climate policies, driving radiative forcing to 8.5 *Wm*^−2^ by the end of the century. The three chosen scenarios represent updates of the previous Representative Concentration Pathways (RCPs) from the CMIP5^15^. These scenarios are fundamental in climate modeling for assessing future climate responses, evaluating risks, and informing mitigation and adaptation strategies under varying socioeconomic and policy conditions.

**Table 1:** CMIP6-GCMs used in our dataset.

Model	Spatial resolution	Country*/*Region	Reference
ACCESS-CM2	250 km	Australia	^41^
ACCESS-ESM1-5	250 km	Australia	^42^
BCC-CSM2-MR	100 km	China	^43^
CAS-ESM2-0	100 km	China	^44^
CESM2-WACCM	100 km	USA	^45^
CMCC-CM2-SR5	100 km	Italy	^46^
CMCC-ESM2	100 km	Italy	^47^
EC-Earth3	100 km	Europe	^48^
EC-Earth3-Veg	100 km	Europe	^48^
EC-Earth3-Veg-LR	100 km	Europe	^48^
GFDL-ESM4	100 km	USA	^49^
MIROC6	250 km	Japan	^50^
MPI-ESM1-2-HR	100 km	Germany	^23^
MRI-ESM2-0	100 km	Japan	^51^
TaiESM1	100 km	Taiwan	^52^

### Pre-Processing of climate models outputs

Reanalysis soil temperature data from 1950 to 2014 included in the ERA5 dataset^16^ were used for bias correction, described in the following section, since there is no reliable ground-based information for the entire country. Reanalysis datasets are widely used in scientific research due to their ability to provide consistent, high- resolution, and long-term records of atmospheric and surface variables^17,18^. Unlike raw observational data, which can be spatially and temporally limited or inconsistent, reanalysis combines observational inputs from various sources—including satellites, weather stations, and radiosondes—with numerical weather prediction models through data assimilation techniques. The ERA5 is the fifth-generation atmospheric reanalysis of the global climate, provided by the European Centre for Medium-Range Weather Forecasts (ECMWF), and offers hourly data at a horizontal resolution of approximately 31 km and includes a wide range of variables relevant to climate and environmental studies^17,19,20^. Its comprehensive coverage and internal consistency make ERA5 particularly suitable for analyzing climate trends, driving hydrological and ecological models, and supporting assessments of climate change impacts on water resources, agriculture, and ecosystems.

In the development of this dataset, we considered soil temperature for three standardized depths consistent with ERA5 reanalysis data: 0.07 m, 0.28 m, and 1.00 m, as our main goal is to enable a wide range of future agricultural studies. Deeper depths were not included since most agricultural root systems do not grow deeper than one meter. Moreover, including other depths would be only possible at the expense of the number of models included, compromising the representativeness and reliability of our multi-model ensemble. As soil temperature outputs from CMIP6 climate models do not typically have standardized soil depths, which do not necessarily correspond to ERA5 depths, it was necessary to interpolate the simulated historical and projections data to match the target ERA5 depths for further bias correction. This was achieved by applying a simple weighted averaging approach, in which the soil temperature values from the climate model layers were combined using a depth-weighted mean^21,22^. The weighting was based on the influence zone of each target depth, approximated by the midpoint of the depth intervals surrounding each ERA5 layer. This methodological step ensures compatibility between datasets while preserving the vertical thermal structure relevant for hydrological and land-surface modeling under climate change scenarios.

Given the coarse spatial resolution of the selected GCMs/ESMs, we performed a follow-up bilinear interpolation to regrid all climate models to a common 0.25°spatial grid in agreement with the ERA5. The bilinear method adopted in regrinding is a well-known method used for its ability to generate a smoother and more continuous representation of spatial data^4,23,24^. This technique utilizes the values of the four nearest grid points surrounding a target point to compute the interpolated value, thereby ensuring that the output grid captures essential spatial variations while maintaining the integrity of the original data’s physical meaning. Bilinear interpolation is especially beneficial for climate data as it minimizes abrupt discontinuities and produces a gradual transition between grid points. We acknowledge that this procedure may severely impact some statistical properties of meteorological variables (e.g.,^4^), so we recommend such consideration of limitation in future use of our dataset.

### Bias correction procedure

Climate models have inherent deficiencies when simulating current climate conditions, triggering systematic errors that propagate into future simulations, especially temperature^25^. Thus, we corrected this systematic bias from the selected climate models using the delta quantile mapping method^26^. This technique explicitly preserves relative and absolute changes between historical and future simulations based on the delta quantile change and detrended quantile mapping methods^27^. In this way, the trends projected by future simulations are maintained after bias correction. The procedure encompasses three steps, in which all future trends are firstly removed from projected quantiles of the soil temperature data series (Eqs. 1 to 3). Then, the bias of the detrended quantiles is corrected using the quantile mapping technique (Eq. 4). In the last step, the projected trends of the quantiles are superimposed on the corrected data series (Eq. 5). Assuming that *o* and *p* are the observed and projected data respectively and that *h* and *f* are the historical and future data, the definition of the non-exceeding probability of the observed historical (*x*_*h*,*o*_) and simulated (*x*_*h*,*p*_) and projected future (*x*_*f*,*p*_) data is computed as:

1 $${P}_{f,p}(t)=F({x}_{f,p}(t))$$

2 $${P}_{h,p}(t)=F({x}_{h,p}(t))$$

3 $${P}_{h,o}(t)=F({x}_{h,o}(t))$$

where *p* and *F* are respectively the probability of non-exceedance associated with a specific value at time t and the empirical cumulative distribution function (ECDF). Thus, a change factor is computed (Eq. 4), associating the historical and future simulations:

4 $$\Delta t={F}_{f,p}^{-1}\left({P}_{f,p}\left(t\right)\right)-{F}_{h,p}^{-1}\left({P}_{f,p}\left(t\right)\right)={x}_{f,p}\left(t\right)-{F}_{h,p}^{-1}\left({P}_{f,p}\left(t\right)\right)$$

where *F*^−1^ is the inverse of the empirical cumulative distribution function (ECDF) and ∆t is the additive correction factor between the simulated quantiles of the historical and future periods. This correction factor preserves absolute changes in the simulated quantiles, recommended for temperature and related variables^26,28^. Finally, the distribution of the simulated data is statistically transformed to conform to the distribution of the observed data, obtaining the simulated historical and future data with the bias correction:

5 $${\hat{x}}_{f,p}\left(t\right)=\Delta \left(t\right)+{F}_{h,o}^{-1}\left({P}_{f,p}\left(t\right)\right)$$

We considered the entire historical period (1950–2015) from ERA5 to correct future projections (2015–2100) of the three considered SSPs using the QDM procedure. Even though this is widely applied in climate change impact assessments, there are some limitations and uncertainties worth mentioning regarding the future use of our bias- corrected product. The QDM generally (*i*) shows high sensitivity to the historical reference data used (ERA5 in our case) for calibration and (*ii*) may be affected by the downscaling process because of the resolution mismatch between the model simulations and observations^9^. Furthermore, we also acknowledge that bias-correction methods are controversial^29,30^ for being unable to retain spatial dependencies^29,31^, possibly producing physically unrealistic outputs and hindering fundamental models’ deficiencies.

## Data Records

This study describes SoilFutures^32^ – a gridded dataset (netCDF) of projected soil temperature with a spatial resolution of 0.25° × 0.25°comprising the Brazilian territory, including both raw and bias-corrected monthly time-series from 15 CMIP6 GCMs as detailed in Table 1. The data spans from the historical period (1950 to 2014) to the end of the century (2015 to 2100), considering a middle-of-the-road (SSP2-4.5), a regional rivalry (SSP3-7.0), and a fossil-fueled development (SSP5-8.5) scenario. Both these 15 bias-corrected individual models and a multi-model ensemble are publicly available under a CC0 license. The data that support the findings of this study are openly available at https://doi.org/10.57760/sciencedb.29673.

## Technical Validation

### Bias correction performance

**Fig. 2:**
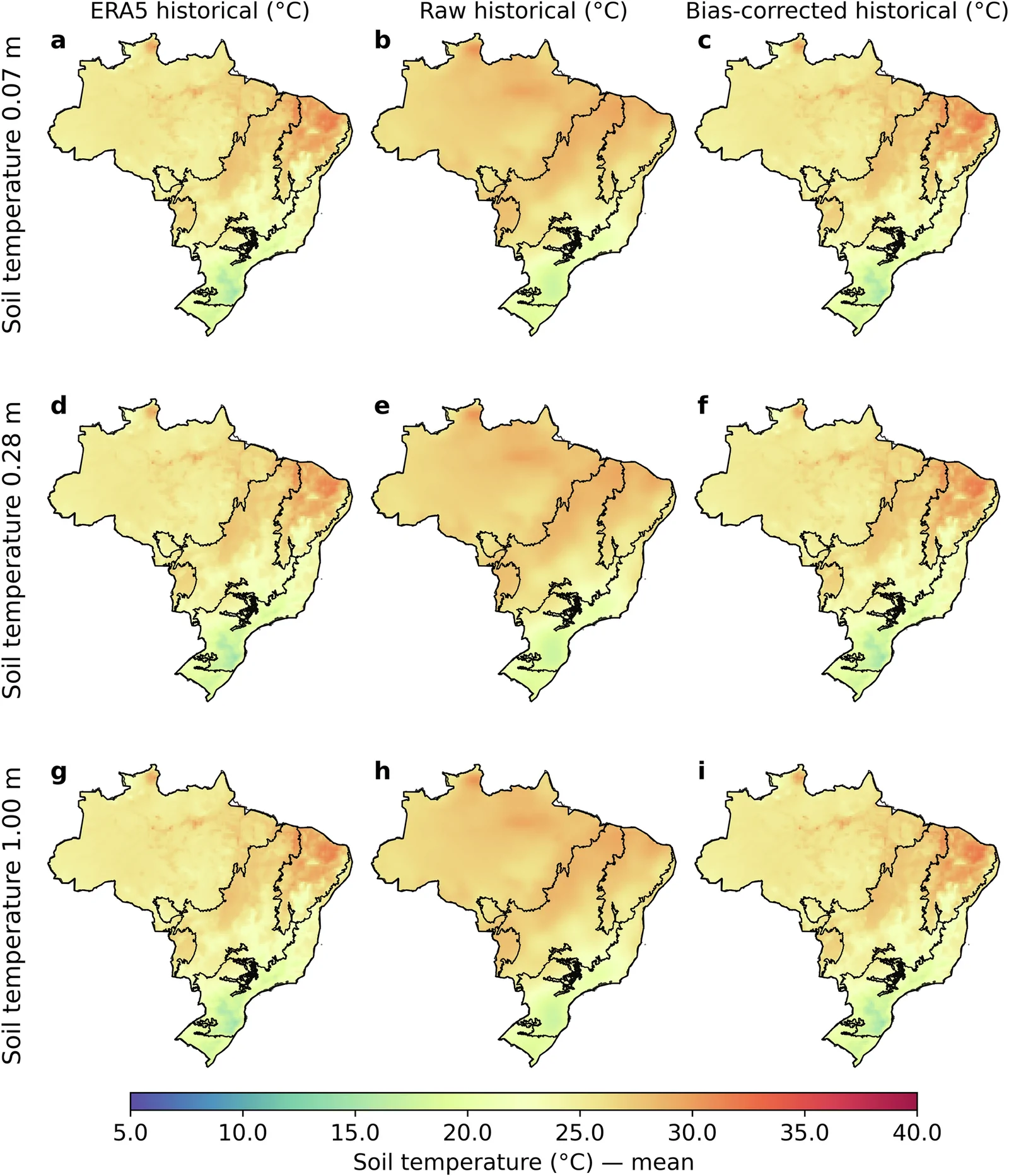
Climatological mean absolute soil-temperature (1980–2014) over Brazil at three depths (0.07 m, 0.28 m, 1.00 m) from raw (raw) and bias-corrected (BC) ensembles means against ERA5. Color scales are ramped (blue = cold temperatures; red = warm temperatures). Thin outlines indicate biome boundaries.

The performance of the bias correction procedure in representing observed soil temperature during the historical period in both each individual and ensemble models will be evaluated. In general, ensembles outperform any individual model since this combination better reflects the inherent uncertainties of each model alone^33^, a reason why we also included it in our dataset. Figure 2 presents the spatial distribution of mean soil-temperature (1980–2014) over Brazil at three depths (0.07 m, 0.28 m, 1.00 m) from raw (raw) and bias-corrected (BC) ensembles means against ERA5, while supplementary material displays values at the 5th and 95th quantiles. The spatial distribution of soil temperature in Brazil is primarily controlled by latitudinal gradients in solar radiation, seasonal atmospheric circulation, and regional land–atmosphere interactions, with higher mean soil temperatures observed in the tropical North and Northeast and lower values in the subtropical South. Superimposed on this macroclimatic pattern, soil thermal regimes are modulated by texture, moisture content, vegetation cover, and land use, which alter heat capacity, thermal conductivity, and surface energy balance, generating significant local heterogeneity. In the Cerrado and Caatinga biomes (Fig. 1), sparse vegetation, sandy to loamy textures, and pronounced dry seasons promote deeper heat penetration and higher seasonal thermal amplitudes, while in the humid South, frequent frontal systems, higher soil moisture, and clay-rich soils enhance thermal buffering, reducing extremes and delaying heat flux propagation.

**Fig. 3:**
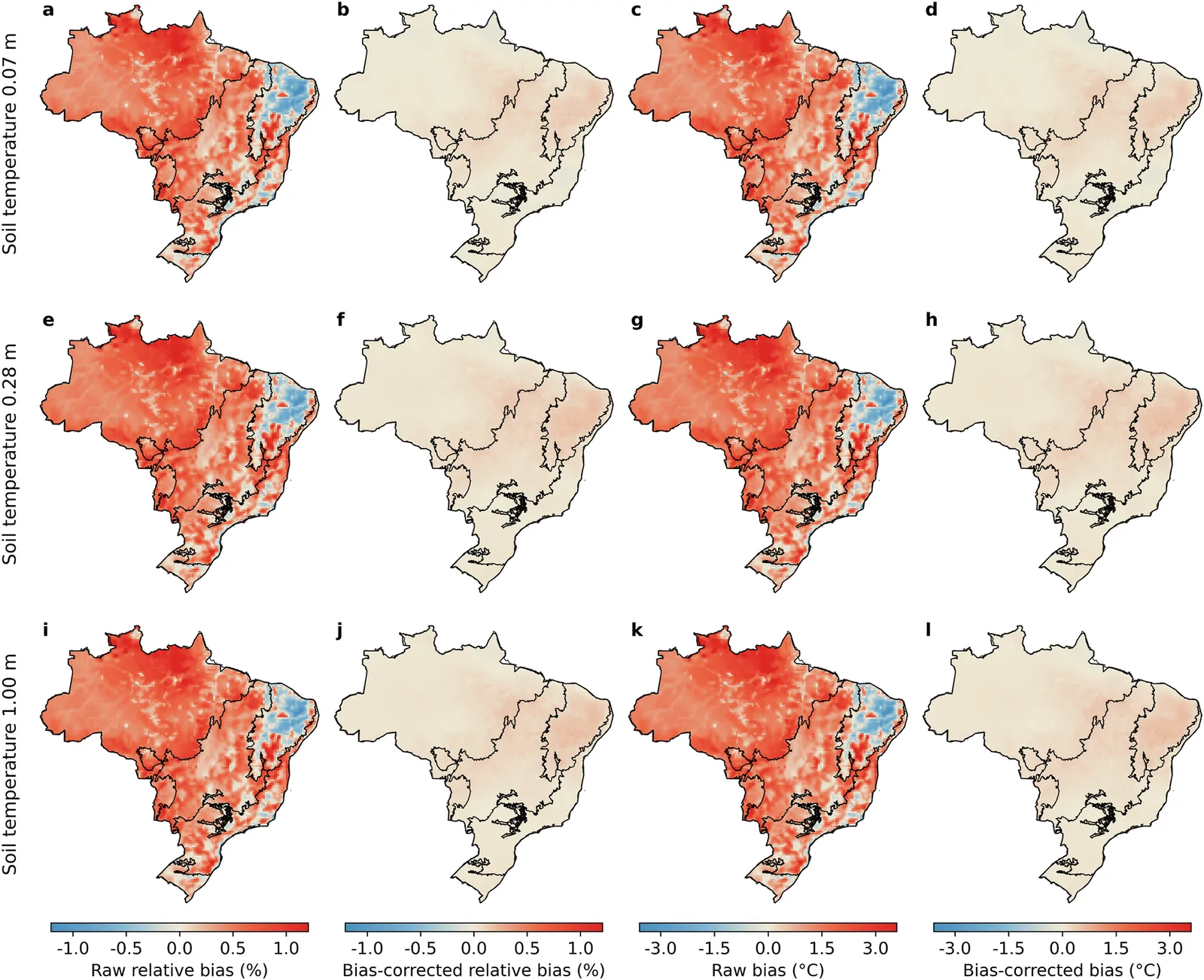
Climatological mean soil-temperature bias (1980–2014) over Brazil at three depths (0.07 m, 0.28 m, 1.00 m), comparing raw (raw) and bias-corrected (BC) ensemble means against ERA5. Columns show: (1) raw relative bias (%), (2) BC relative bias (%), (3) raw absolute bias (^°^C), and (4) BC absolute bias (^°^C). Color scales are diverging and centered at zero (blue = model colder than ERA5; red = warmer). Thin outlines indicate biome boundaries.

To obtain a first-order view of bias correction performance, we computed the relative and absolute bias between long-term (1980–2014) means from the CMIP6 models and the ERA5 reanalysis, contrasting raw against bias-corrected records. Relative bias was calculated as the difference between the model and reference dataset (ERA5) divided by the reference value and expressed as a percentage, while absolute bias corresponds to the direct difference between model and reference values in the original units. Both raw and bias-corrected multimodel ensemble mean overall overestimate long-term soil temperature at all three depths, as shown in Fig. 3, with much warmer deviations (up to + 3 °C) exhibited by the raw ensemble mean (plots c, g, and k). Specifically in the Caatinga Biome, the raw ensemble presents a cold bias of up to −3 °C, which is attenuated in the bias-corrected ensemble, now presenting a slightly warmer bias compared to the other biomes. After bias correction, residual biases were reduced to near zero despite the slight warm bias in the Cerrado-Amazonia transition and Caatinga. The cold bias observed in the raw outputs over the Caatinga—an arid to semi-arid biome—has been associated in CMIP6 land-surface schemes with deficiencies in latent heat flux, surface downwelling shortwave radiation, and surface albedo parameterizations. This negative bias relative to ERA5 likely reflects the region’s strong climatic variability, low evapotranspiration, reduced gross primary production, sparse vegetation cover, and widespread land degradation, which make the surface energy and water balances particularly difficult for models to represent accurately^34,35^.

**Fig. 4:**
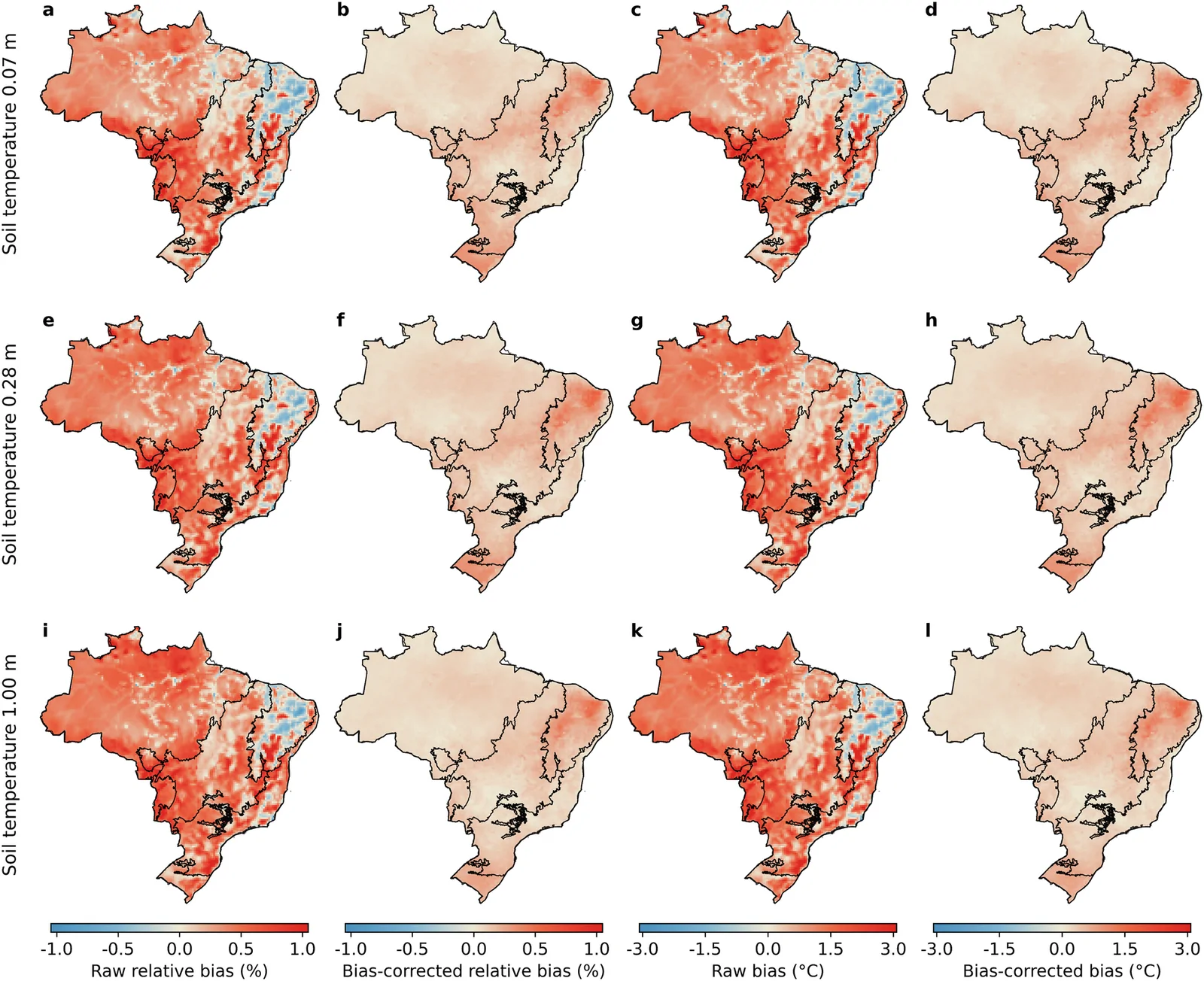
Climatological 5th soil-temperature bias (1980–2014) over Brazil at three depths (0.07 m, 0.28 m, 1.00 m), comparing raw (raw) and bias-corrected (BC) ensemble means against observations. Columns show: (1) raw relative bias (%), (2) BC relative bias (%), (3) raw absolute bias (^°^C), and (4) BC absolute bias (^°^C). Color scales are diverging and centered at zero (blue = model colder than observations; red = warmer). Thin outlines indicate biome boundaries.

We also evaluated the high and low extremes of the long-term distribution using the 5th and 95th percentiles. The spatial distribution of cold and warm bias with respect to the 5th percentile (Fig. 4) is mostly consistent with what was observed in the long-term means in both raw and bias-corrected datasets. When analyzing the raw multimodel ensemble, a cold bias is also observed in some scatter areas in the Amazonia and Mata Atlântica biomes. As expected, the bias correction reduced these biases with a final overall warm bias across the country, especially in Caatinga, as mentioned above for the long-term mean. It is worth noting that, in terms of low extremes, the bias-corrected ensemble presents generally warmer relative and absolute biases, compared to when we look at the long-term means. Future users of this dataset should take this into consideration, especially when using this data for assessing projected extreme low temperatures in Northeast and South Brazil. The magnitude of bias is similar and spatially coherent across soil depths in both raw and bias-corrected datasets.

**Fig. 5:**
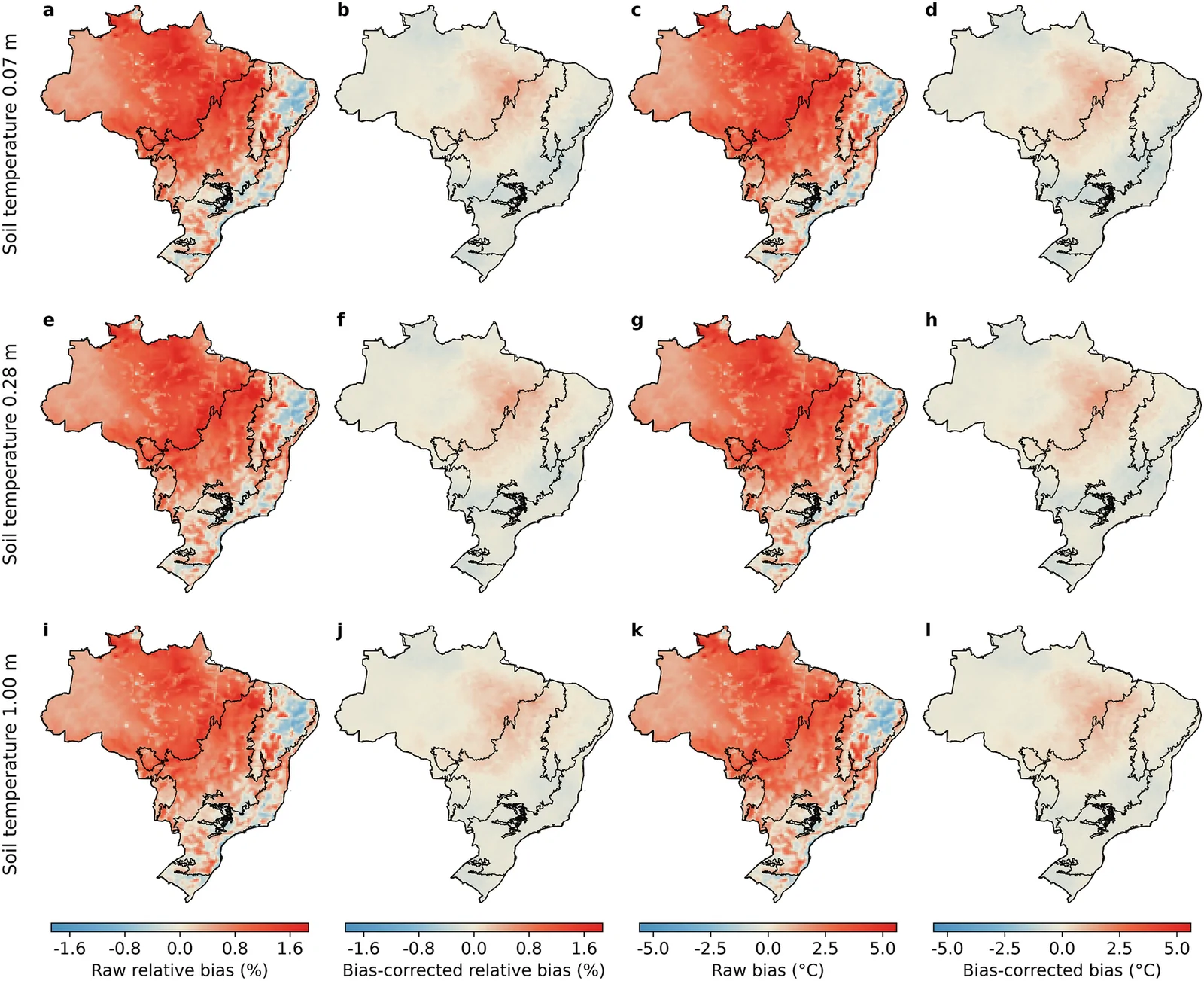
Climatological 95th soil-temperature bias (1980–2014) over Brazil at three depths (0.07 m, 0.28 m, 1.00 m), comparing raw (raw) and bias-corrected (BC) ensemble means against ERA5. Columns show: (1) raw relative bias (%), (2) BC relative bias (%), (3) raw absolute bias (^°^C), and (4) BC absolute bias (^°^C). Color scales are diverging and centered at zero (blue = model colder than observations; red = warmer). Thin outlines indicate biome boundaries.

When considering the high extreme temperatures (95th percentile; Fig. 5) the bias-corrected ensemble exhibits warm bias in the Cerrado-Amazonia biomes transition, as regarded in the long-term means and 5th percentiles, as well as cold bias in the North portion of the Amazonia and South portions of Cerrado, Caatinga, and Pampa biomes. These warm biases are typically within ∼1.6% and decrease with depth, and their spatial patterns becomes smoother, consistent with subsurface thermal filtering. We point out that this warm bias in the Cerrado-Amazonia transition corresponds to the so-called Amazon Arc of Deforestation, an area with increased native land cover change to agricultural uses, which exacerbates compound drought-high temperatures in the region^36^. The reported land conservation in this region may impose limitations to land-atmosphere coupling in the global climate models and further bias correction using reanalysis products, such as ERA5^37^.

Overall, the QDM substantially improved the historical simulations, reducing systematic bias in both long- term means and extremes across all depths, with a clear depth-dependent improvement. After correction, relative biases are largely confined to [−1.6%, +1.6%] and cluster around zero, indicating that QDM addressed not only the mean offset but also quantile-dependent errors in the raw GCM/ESM distributions.

### Performance in reproducing intra-annual variability

We also compared the performance of the bias-corrected and raw ensemble means in reproducing the long-term soil temperature seasonality over Brazil. Because soil temperature is primarily climate-driven at monthly scales, we also stratified our analysis by using the Koppen–Geiger climate classes to separate hydroclimatic regimes (e.g., radiation and precipitation regimes) while avoiding confounding effects of heterogeneous and time-varying land cover, such as biomes and geographic regions. For the six most widespread climate zones in Brazil, we computed area-weighted long-term monthly means based on the observations and the raw and bias-corrected ensemble means.

**Fig. 6:**
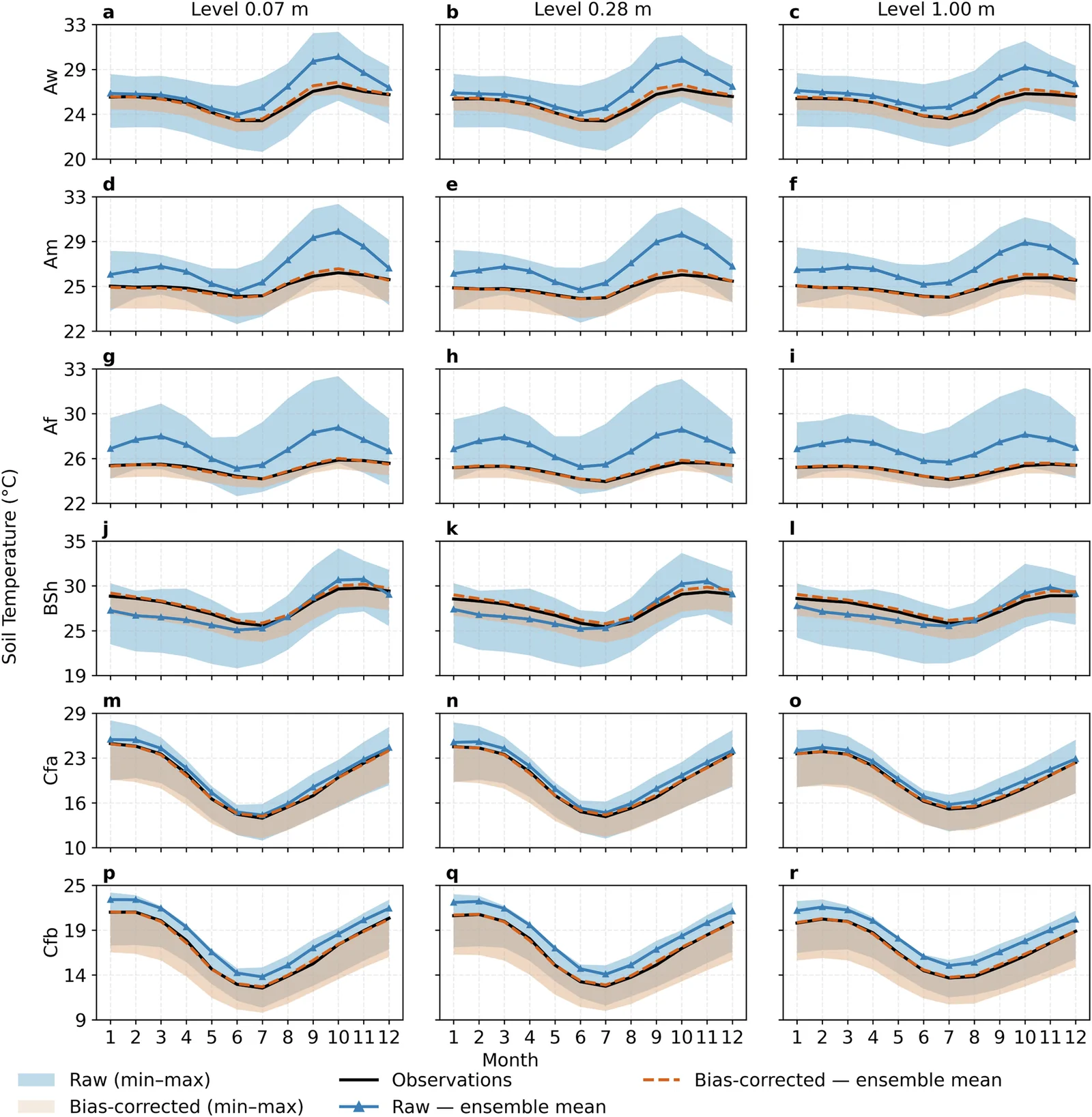
Intra-annual variability in the long-term monthly soil temperature across depths in each Koppen-Geiger climate zone: Tropical with a dry Winter season (Aw, **a**-**c**), humid and sub-humid (Am, **d**-**f**), and humid without a dry season (Af, **g**-**i**); hot Semiarid (BSh, **j**-**l**); Subtropical with a hot Summer season (Cfa, **m**-**o**); and Temperate with a mild Summer season (Cfb, **p**-**r**). Long-term monthly means are displayed in blue for the raw, in red for the bias-corrected, and black for the ERA5 values. Shaded bands represent the maximum-minimum range of raw and bias-corrected means considering all models composing the ensemble.

The intra-annual cycle reflects, at the superficial soil layer, the expected physical controls: the peak–to–trough variations set by insolation and the north–south gradient in solar forcing. In the humid tropics (Aw, Am, and Af) the amplitude is damped, but not so in the humid subtropics (Cfa, Cfb) (Fig. 6, plots a-i). For the Tropical climate zones (A-), the highest soil temperatures are observed during the austral Spring (September-November), which corresponds to the usual characteristic higher air temperatures in these climate zones. This elevated soil temperatures across all soil depths is even more pronounced in the Semiarid climate (BSh) during that season. Compared to the bias-corrected values, the raw ensemble presents much warmer bias and larger variability (depicted by the light-blue max-min bands), especially in the Tropical climate zones. In the BSh, we noted a shift in the intra-annual direction of bias, with a cold bias during the Summer and Fall seasons (January-June, plots j-l in Fig. 6) and a slightly warmer bias during the austral Spring. This is consistent with the tendency of climate models to exhibit cold biases over semiarid/arid lands.

In terms of long-term monthly means, the bias correction was able to remove the systematic bias in the raw models, while preserving the phase of the seasonal intra-annual cycle. Improvements are most noticeable in the Tropical climates (A-), in which the raw values showed larger deviations from ERA5 values and variability. Specifically, in Af and Am, where the observed seasonal signal is weak, the bias correction mainly eliminates a quasi-constant warm offset. In terms of variability among bias-corrected models, the bias correction procedure yielded negative deviations from the ERA5 observations across all climate zones, specially producing wider bands below the bias-corrected ensemble means due to large disagreement among the models in the Subtropical and Temperate climates (Cfa and Cfb in Fig. 6, plots m-r). Both means and max-min intra-annual variation behave alike across soil depths.

### Future soil temperature pathways

Our dataset includes projections extending through the end of the 21st century, allowing us to explore projected changes in soil temperature under potential future climate conditions. For analytical conciseness, the soil temperature results presented in this main text are relative to the superficial soil layer (0–0.07 m) and based on the projected changes in the distant future period (2070–2100) for all Shared Socioeconomic Pathways (i.e., SSP2- 4.5, SSP3-7.0, and SSP5-8.5). As the order and magnitude of projected changes in the other deeper soil layers are consistent with those in the superficial layer, these other results are depicted in the Supplementary Material. Thus, the analyses covered here for the most superficial depth also relate to the other soil depths.

**Fig. 7:**
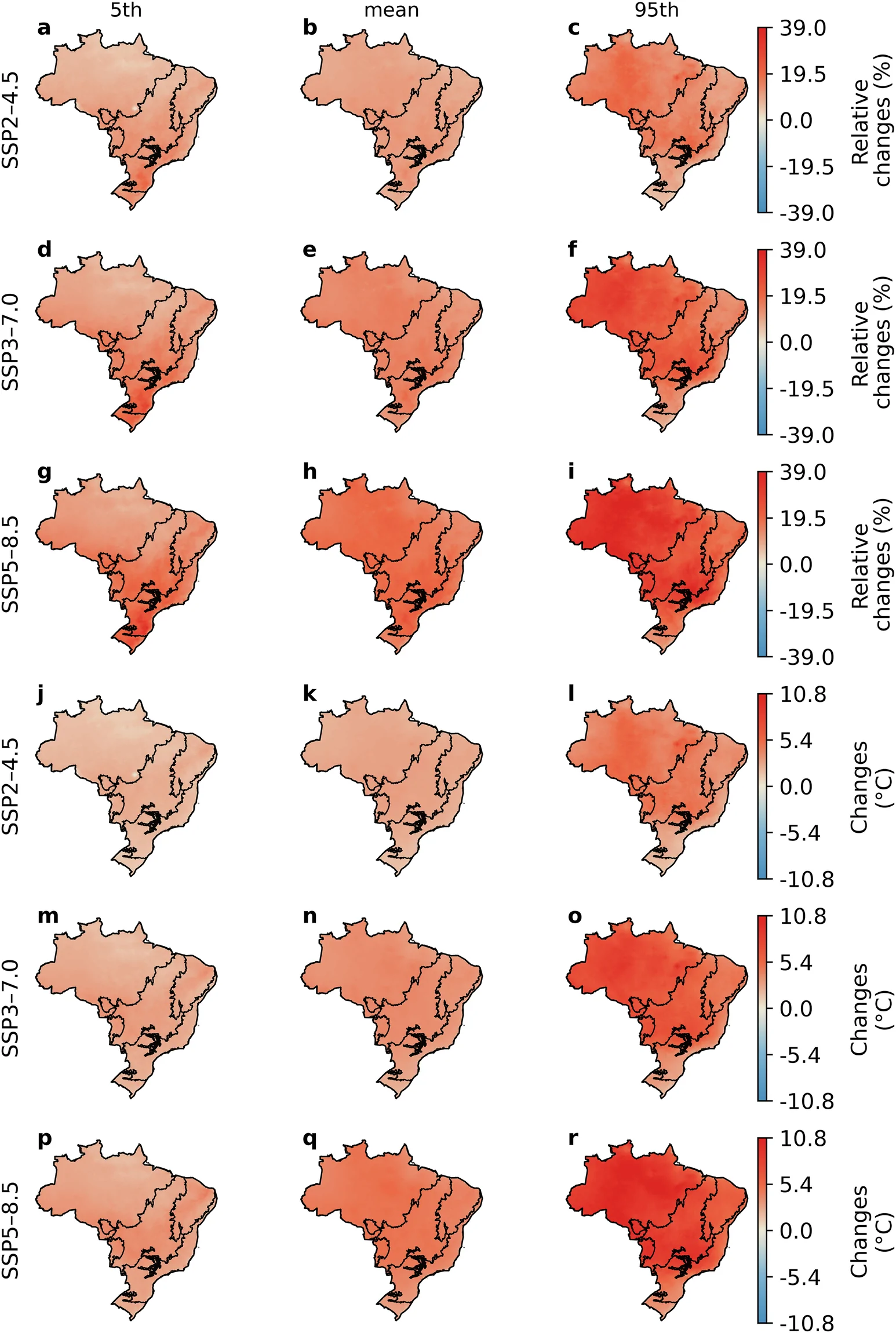
Relative and absolute changes in the long-term mean and extreme (5th and 95th percentiles) values of soil temperature at superficial soil layer (0–0.7 m) between the historical (1980–2014) and distant future (2070–2100) periods based on the bias-corrected multimodel ensemble.

Across all scenarios, soil temperature is expected to increase in the distant future all over Brazil, mainly in the most pessimistic scenario (SSP5-8.5) with an average increase of 4.30 °C (Fig. 7, plot q). In terms of long- term means, the projected increase in soil temperature is homogeneous over the territory, with negligible differences among biomes. Projected increase in extreme temperatures differs, on the other hand, among biomes. Larger increments in extreme low soil temperatures compared to the historical period are expected in the southern portion of Brazil, consistent across SSPs and more noticeable in the SSP5-8.5, with an average increase of 2.51 °C in the Pampa biome (Fig. 7, plots a, d, and g). On the other extreme end, future high soil temperatures are expected to significantly increase in the North and Central regions of Brazil. The most optimistic scenario (SS2-4.5) projected an increase of 2 °C in these two biomes, homes of important agricultural activities and environmental conservation that may face future disruptions due to these changes in soil temperature. Again, the SSP5-8.5 presents the largest increments in soil temperature of up to + 5 °C over the Amazon and Cerrado biomes (Fig. 7, plot r). This most pessimistic trajectory indicates an increase in extreme high soil temperatures of 10°(≈ 40% relative change) C in some locations in these two biomes. It means having a superficial soil layer with up to 40 °C by the end of 2100 in SSP5-8.5. Overall, the results indicate an upward shift of the entire quantile distribution, not only the extremes. These findings suggest potential risks for soil moisture retention, microbial activity, and crop productivity, especially in the Cerrado, where agriculture is an important economic sector.

We summarize how late-century warming reshapes the vertical structure of soil temperature across Brazilian biomes (Supplementary Fig. 10). Across scenarios, the profiles shift upward at all depths from the historical baseline to the late-century period (Supplementary Fig. 10a). In the Cerrado, Amazon, and Atlantic Forest, surface warming is slightly larger than subsurface warming (Supplementary Fig. 10b). In contrast, the Pampa and Caatinga show positive ∆*g* across layers, implying a flatter or weakly inverted profile in which deeper soils warm comparably to (or more than) the surface. Overall, changes in ∆*g* are small relative to the mean warming, but they reveal a tendency toward near-surface amplification that is different across biomes.

**Fig. 8:**
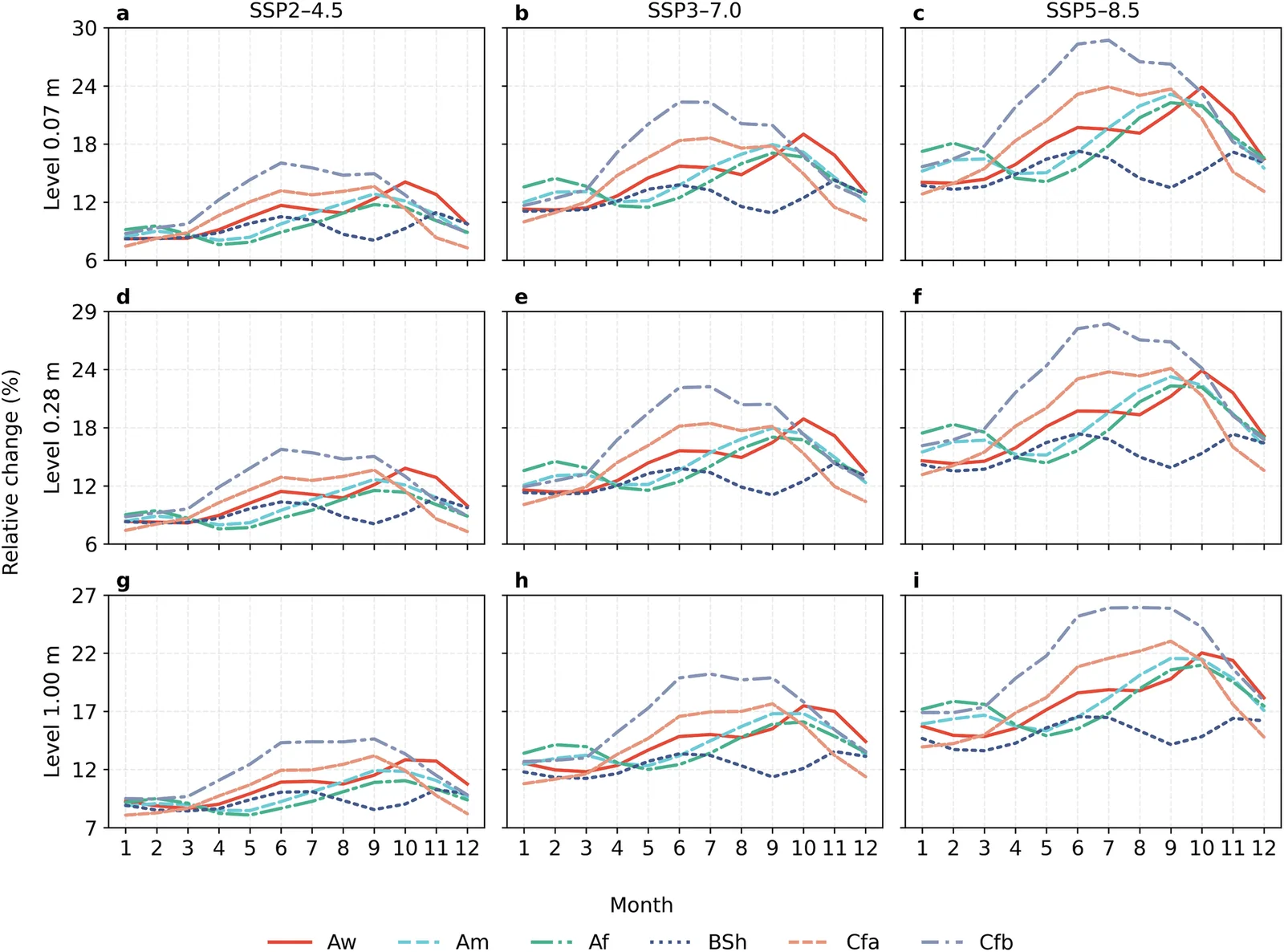
Relative and absolute intra-annual changes in long-term monthly soil temperatures at all three soil depths (0.07, 0.28, and 1.00 m, row-wise) between the historical (1980–2014) and distant future (2070–2100) periods. These changes are based on the bias-corrected multimodel ensemble dataset, considering the SSP2-4.5, SSP3-7.0, and SSP5-8.5 scenarios (column-wise).

We also analyzed the climate change impacts on the long-term monthly soil temperature in the distant future for capturing seasonality nuances based on intra-annual variability. Significant increases in the intra-annual monthly temperature are also expected, with as low as 6% and as high as 30% relative increments considering the most optimistic (SSPT2-4.5) and pessimistic (SSP5-8.5) scenarios (Fig. 8). The order of magnitude of these relative changes between the historical and distant future periods is alike in all three soil depths. Overall, the Subtropical and Temperate climates are projected to face the largest increases in monthly soil temperatures, especially from the austral Fall and Winter (April-September) (dark blue, orange, and red lines in Fig. 8), reaching relative changes of up to 30% at the superficial soil layer for the SSP5-8.5. Conversely, the Tropical climates (red, light blue, and green lines in Fig. 8) present more substantial increases mainly during the austral Spring (September-December), reaching a relative change of 24% at the superficial layer for the SSP5-8.5.

## Usage Notes

This study describes a dataset containing downscaled bias-corrected soil temperature data from 1950 to 2100 for Brazil SoilFutures-BR^32^, with a high spatial resolution of 0.25°available in NetCDF under an open CC0 license. We provide the data from each of the 15 individual GCMs/ESMs (see Table 1), as well as from a derived multi-model ensemble, to cover a wide range of applications by researchers and practitioners working in the interface of soil and climate sciences. Having this in mind, the projected changes in soil temperature are based on three SSPs from a mid to the highest emission trajectory (SSP2-4.5, SSP3-7.0, and SSP5-8.5) and also include the raw simulations from all models.

The availability of this gridded soil temperature dataset offers significant benefits for scientific research in both climate, environment, and agriculture fields. By providing consistent, bias-corrected data across historical and future periods, it enables more accurate assessments of soil thermal regimes under changing climate and socioeconomic scenarios. In environmental studies, this dataset supports improved modeling of land–atmosphere interactions, hydrological processes, and ecosystem dynamics. In agriculture, soil temperature is a key variable influencing productivity and sustainability, along with variables such as moisture dynamics, seed germination, root development, and microbial activity. Additionally, unsustainable agricultural practices progressively erode both current soil conditions and the trajectory of their future functioning, thereby undermining soil health. Intensive input-driven systems and conventional practices such as heavy tillage, monocropping, excessive chemical fertilizers and pesticides, and poor residue management accelerate the loss of soil organic matter, intensify erosion, compaction, salinization, acidification, and contamination, and sharply reduce soil biodiversity. Over time, these processes compromise climate resilience by depleting soil carbon stocks and increasing greenhouse gas emissions, locking soils into degraded states that are costly or impossible to fully restore. Our SoilFutures-BR dataset^32^ provides an adequate spatial resolution for crop modeling, agricultural planning, and climate adaptation strategies from a soil-plant-atmosphere improved understanding.

### Uncertainties and limitations

Despite its broad applicability, the dataset also presents some limitations that users should consider. The use of raw model outputs may introduce systematic biases and may not accurately capture local or regional climate variability, especially in areas with complex topography or land surface characteristics (1). ERA5 provides a consistent, spatially continuous, and temporally homogeneous gridded product that captures the large-scale seasonal cycles and long-term variability of soil temperature^38^, making it a suitable reference for bias correction despite the country’s strong pedological heterogeneity and the absence of a national *in situ* soil temperature monitoring network with adequate spatial and temporal coverage. However, ERA5 is a model-based reanalysis dataset that combines numerical modeling with data assimilation and therefore does not represent purely observational data (2), which introduces an additional layer of structural uncertainty and a source of potential propagation^9,38,39^. Moreover, ERA5 exhibits biases that vary in magnitude and spatial distribution—such as cold biases in maximum temperatures and warm biases in minimum temperatures—which can influence simulated trends and variability and must be considered when interpreting the projections. Although bias correction techniques have been applied, individual model outputs may still retain uncertainties arising from the statistical methods used, including assumptions about the stationarity of biases and the distribution of variables (3). Due to not only the spatial resolution relative to micro-scale processes but also uncertainties related to the SSP assumptions, users are advised not to interpret results at the pixel level or for individual years (4). Soil temperature is directly linked to local-scale variability related to soil properties and land cover and land use, which both were not considered at actual version of the dataset produced (5). As we shaped our discussion around the bias correction performance, projections should be carefully used to investigate possible future changes and trends in long-term metrics, typically 30 years, following recommendations from the World Meteorological Organization for meaningful interpretations.

## Supplementary information

Supplementary Information

## Data Availability

SoilFutures Dataset comprises 15 CMIP6 General Circulation Models (GCMs) bias-corrected individual models and a multi-model ensemble of soil temperature under climate change (SSP2-4.5, SSP3-7.0, and SSP5-8.5 scenarios) for the Brazilian territory under the under a CC0 license. The data that support the findings of this study are openly available at https://doi.org/10.57760/sciencedb.29673.

## Code availability

Pre- and post-processing tasks were carried out using the R-packages of the Climate4R project, extensively described in^40^. This framework was developed to address the needs of different climate-impact studies and includes a roll of R-packages to access, pre- and post-process, and visualize climate data. All the packages and documentation, including tutorials and example-notebooks, are available through the following Github link: https://github.com/SantanderMetGroup/climate4R.
