# Supplementary material for: SoilFutures-BR: Bias-Corrected Soil Temperature Projections for Brazil
Source: Sci Data. 2026 Jun 25;13:1417. doi: 10.1038/s41597-026-07500-z (PMC13661618; doi:10.1038/s41597-026-07500-z)
Supplement: Supplementary file 1 [file 41597_2026_7500_MOESM1_ESM.docx]

**SUPPLEMENTAL MATERIAL - S1**

Figure 1: Climatological 5th soil temperature (1980–2014) over Brazil at three depths (0.07 m, 0.28 m, 1.00 m) from raw (raw) and bias-corrected (BC) ensembles means against ERA5. Color scales are ramped (blue = cold temperatures; red = warm temperatures). Thin outlines indicate biome boundaries.

**SUPPLEMENTAL MATERIAL – S2**

Figure 2: Climatological 95th soil temperature (1980–2014) over Brazil at three depths (0.07 m, 0.28 m, 1.00 m) from raw (raw) and bias-corrected (BC) ensembles means against ERA5. Color scales are ramped (blue = cold temperatures; red = warm temperatures). Thin outlines indicate biome boundaries.

**SUPPLEMENTAL MATERIAL – S3**

Figure 3: Spatial distribution of soil temperature at 0.28 m depth across Brazil for the historical baseline period (1985–2014) and projected future scenario SSP2-4.5 (2071–2100) after bias correction. Columns show historical values (left), future projections (middle), and projected changes (right). Rows represent the mean (top), 5th percentile (middle), and 95th percentile (bottom).

**SUPPLEMENTAL MATERIAL – S4**

Figure4: Spatial distribution of soil temperature at 1.0 m depth across Brazil for the historical baseline period (1985–2014) and projected future scenario SSP2-4.5 (2071–2100) after bias correction. Columns show historical values (left), future projections (middle), and projected changes (right). Rows represent the mean (top), 5th percentile (middle), and 95th percentile (bottom).

**SUPPLEMENTAL MATERIAL – S5**

Figure 5: Vertical soil temperature structure and its late-century change by biome. (a) Biome-mean temperature profiles (°C) at 0.07, 0.28, and 1.00 m for the historical baseline (1980–2014; Hist) and the future (2070–2100; Fut) under SSP5–8.5. (b) Mean change in vertical gradient, ∆g = g_Fut_ − g_Hist_ (°C.m^−1^), shown for near-surface (0.05–0.28 m), mid–deep (0.28–1.00 m), and surface–deep (0.05–1.00 m) layers. Positive ∆g implies a flatter or inverted vertical profile; negative ∆g implies a steeper negative gradient (surface warming more than deep).
